# Supplementary material for: Bactericidal Activity of Ceragenin in Combination with Ceftazidime, Levofloxacin, Co-Trimoxazole, and Colistin against the Opportunistic Pathogen Stenotrophomonas maltophilia
Source: Pathogens. 2022 May 26;11(6):621. doi: 10.3390/pathogens11060621 (PMC9227598; doi:10.3390/pathogens11060621)
Supplement: Supplementary file 1 [file pathogens-11-00621-s001.zip › pathogens-1734661-supplementary.pdf]

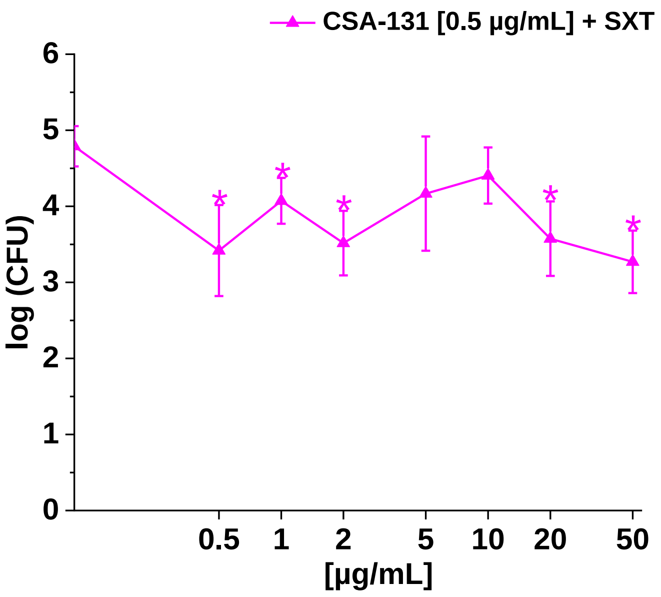

Figure S1. Killing activities of CSA-131 and SXT studied at with 0.5 and 0.5-50 µg/mL respectivelly, against *S. maltophilia* strain 1 was determined using a standard colony counting assay. Results show mean±SD from six measurements. * indicates statistical significance at ≤0.05, ** ≤0.01, and *** ≤0.001.

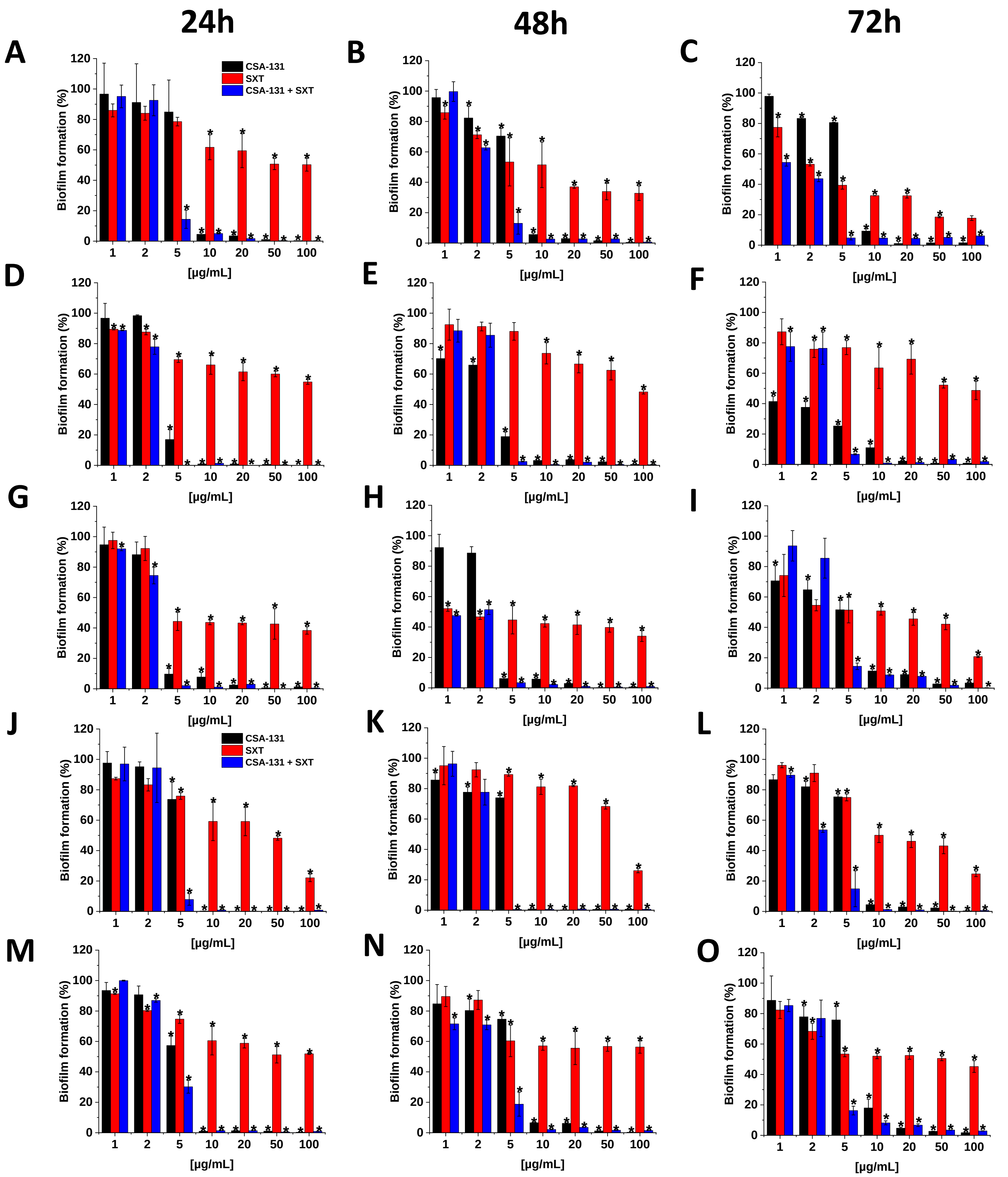

Figure S2. Prevention of biofilm formation by *S.maltophilia* strain 1 (panels A-C), strain 2 (panels D-F), strain 3 (panels G-I) strain 4 (panels J-L), strain 5 (panels M-O) during treatment with CSA-131, SXT, and CSA-131+SXT. Formation of biofilm in the presence of tested compounds at concentration ranging 1–100 µg/mL was assessed using the resazurin-based fluorimetric method after 24, 48, and 72 hours incubation. Results show mean±SD from 3–6 measurements. * indicates statistical significance ≤0.05, ** ≤0.01, and *** ≤0.001

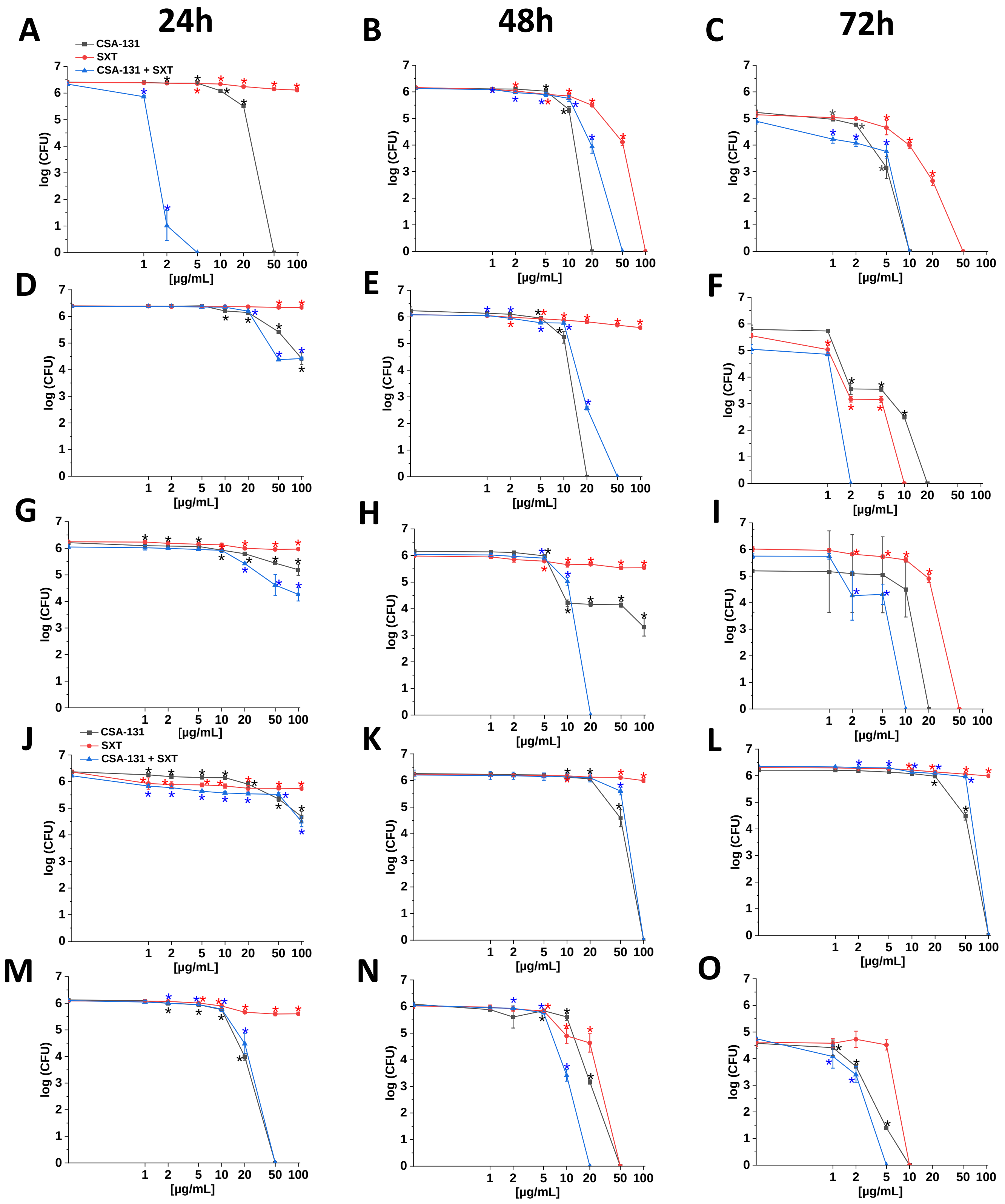

Figure S3. Disruption of the biofilms formed by *Stenotrophomonas maltophilia* strain 1 (panels A–C), strain 2 (panels D-F), strain 3 (panels G-I) 4 (panels J-L) and strain 5 (panels M-O), upon traetment with CSA-131, SXT, and CSA-131+SXT. Bacteria outgrow at 24, 48, and 72 hours from biofilm treated with testeted agents at concentration 1–100 µg/mL. Results show mean±SD from 3–6 measurements. * indicates statistical significance ≤0.05, ** ≤0.01, and *** ≤0.001.
